# MicroRNA-510 mediated negative regulation of Caveolin-1 in fibroblasts promotes aggressive tumor growth

**DOI:** 10.3389/fimmu.2023.1116644

**Published:** 2023-09-25

**Authors:** Brooke King, Bradley A. Krisanits, Qi J. Guo, Bobbie Blake, Lourdes M. Nogueira, Gurbani Jolly, Arabia Satterwhite, David P. Turner, Stanley Hoffman, Ashley Evans-Knowell, Victoria J. Findlay

**Affiliations:** ^1^ Department of Pathology & Laboratory Medicine, Medical University of South Carolina, Charleston, SC, United States; ^2^ Department of Surgery, Massey Cancer Center, Virginia Commonwealth University, Richmond, VA, United States; ^3^ School of Medicine, Virginia Commonwealth University, Richmond, VA, United States; ^4^ Department of Biological and Physical Sciences, South Carolina State University, Orangeburg, SC, United States; ^5^ Division of Rheumatology, Department of Medicine, Medical University of South Carolina, Charleston, SC, United States

**Keywords:** Caveolin-1, microRNA, breast cancer, disparities, stroma

## Abstract

**Introduction:**

In the US, despite the recent decline in breast cancer deaths, a persistent mortality disparity exists between black and white women with breast cancer, with black women having a 41% higher death rate. Several studies are now reporting that racial disparities can exist independent of socioeconomic and standard of care issues, suggesting that biological factors may be involved. Caveolin-1 (Cav1) loss in the tumor stromal compartment is a novel clinical biomarker for predicting poor outcome in breast cancer including triple negative subtype, however the mechanism of Cav1 loss is unknown. We previously identified miR-510-5p as a novel oncomir and propose here that the high levels observed in patients is a novel mechanism leading to stromal Cav1 loss and worse outcomes.

**Methods:**

Cav1 was identified as a direct target of miR-510-5p through luciferase, western blot and qPCR assays. Stromal cross talk between epithelial cells and fibroblasts was assessed *in vitro* using transwell co-culture assays and in vivo using xenograft assays.

**Results:**

We found that Cav1 is a direct target of miR-510-5p and that expression in fibroblasts results in an ‘activated’ phenotype. We propose that this could be important in the context of cancer disparities as we also observed increased levels of circulating miR-510-5p and reduced levels of stromal Cav1 in black women compared to white women with breast cancer. Finally, we observed a significant increase in tumor growth when tumor cells were co-injected with miR-510-5p expressing cancer associated fibroblasts *in vivo*.

**Conclusion:**

We propose that miR-510-5p mediated negative regulation of Cav1 in fibroblasts is a novel mechanism of aggressive tumor growth and may be a driver of breast cancer disparity.

## Introduction

Breast cancer remains a worldwide health issue as it remains the leading cause of cancer in women and the second leading cause of cancer related death. Recent statistics from the American Cancer Society show that black women have about a 40% higher mortality rate than white women despite a slightly lower incidence of the disease. We are also beginning to understand that biological factors of the tumor and microenvironment play a role in driving cancer disparities (1–3). However, the biological factors that contribute to the disparities in breast cancer mortality that we observe are not well understood.

Caveolin-1 (Cav1) is a scaffolding protein with multiple cellular functions. In breast cancer, Cav1 has been shown to be strongly associated with clinical outcomes. Specifically, Cav1 loss in the stromal compartment predicts poor clinical outcome, including early tumor recurrence, tamoxifen-resistance lymph node metastasis, and poor survival (4, 5). Overall, breast cancer patients show a 60% reduction in 5-year survival rates with a loss of stromal Cav1 compared to patients with high stromal Cav1 expression. In triple negative breast cancer (TNBC) patients, the 5-year survival rate is 75.5% for high stromal Cav1 versus 9.4% for absent stromal Cav1. A loss of stromal Cav1 also predicts progression to invasive disease in DCIS (ductal carcinoma *in situ*) patients, suggesting that a loss of Cav1 regulates tumor progression (6). Stromal loss of Cav1 expression was a better prognostic factor for overall survival in TNBC than tumor size, histological grade, p53 and Ki67 status. Interestingly, no similar prognostic effect of tumor epithelial Cav1 has been demonstrated on overall survival or with AJCC stage, T status, lymph metastasis, distant metastasis and histological grade. Epithelial and stromal Cav1 expression were also found to be inversely correlated suggesting that the role of Cav1 may be biphasic in the progression of breast cancer (7).

The tumor microenvironment is known to be a strong factor driving tumor progression. The recruitment and/or activation of fibroblasts leads to aggressive tumor growth. Studies have shown that cancer associated fibroblasts (CAFs) isolated from breast cancer patients have a molecular profile similar to that from Cav1-/- mammary stromal cells suggesting that the molecular loss of stromal Cav1 in breast cancer patients is a key factor leading to aggressive tumor growth (8, 9). Understanding the mechanism of stromal Cav1 loss is therefore essential to improve outcome in women with aggressive disease. Of note, in CAFs with Cav1 protein loss, studies found that the transcript levels of Cav1 either increased or remain the same, suggesting a potential microRNA mediated mechanism of regulation (10).

This study is the first to describe a mechanistic link between microRNA-510-5p (miR-510-5p) expression in epithelial cells and Cav1 loss in fibroblasts. We provide data supporting a role for miR-510-5p in the activation of fibroblasts leading to aggressive tumor growth and cellular migration and propose this as a model contributing to breast cancer disparities.

## Materials and methods

### Human samples

Upon IRB approval, de-identified serum samples were obtained from the Hollings Cancer Center Tissue Biorepository at the Medical University of South Carolina. Serum samples consisted of a cohort of 25 samples: 19 patients [9 African American (Grade 1 n=1; Grade 2 n=2; Grade 3 n=5; Grade unknown n=1), 10 Caucasian American Grade 1 n=2; Grade 2 n=2; Grade 3 n=6)] with breast cancer and 6 patients (3 African American, 3 Caucasian American) with benign breast disease.

### Cell culture and reagents

Cell lines (MDA 231, NIH3T3, WPMY1 and HEK293) were cultured in DMEM supplemented with 10% fetal bovine serum and 100 U of penicillin/streptomycin (P/S) and maintained at 37°C with 5% CO2. All cell lines were obtained from ATCC. All tissue culture reagents were purchased from Invitrogen (Carlsbad, CA).

The animal study was reviewed and approved by the Institutional Animal Care and Use Committee at the Medical University of South Carolina. Approval # ARC-2907.

### Xenograft assay (*in vivo*)

#### CAF isolation

MDA 231 breast cancer cells (1 x 10^6^) either stably expressing miR-510-5p or scrambled control were orthotopically injected into 8 week old female nude mice and allowed to grow until the tumors reached a size of ~1500 mm^3^ at which point tumors were harvested for CAF isolation.

#### Experiment 1

MDA 231 cells (5 x 10^4^) and CAFs (1.5 x 10^5^) isolated from either miR-510-5p expressing or scrambled control tumors were co-injected orthotopically into 6 week old nude mice; n=8 per group. Tumors were measured bi-weekly with electronic calipers and tumor volume calculated using the formula (L x W^2^)/2. Orthotopic injections of either 2 x 10^5^ MDA 231 cells, miR-510-5p CAFs or scrambled CAFs alone served as additional controls.

### CAF isolation

Tumors were minced before digestion in a 0.5mg/ml Collagenase I (Sigma) solution in αMEM for 35 minutes at 37°C with agitation. Next, the solution was vortexed for 30 seconds before filtration through a 40µM cell strainer and centrifugation at 1600 rpm for 5 minutes. The pellet is resuspended in 5ml 0.1% BSA/PBS, centrifuged at 1600rpm for 5 minutes and the resultant pellet resuspended in 20% FBS/αMEM + P/S and plated. 48 hours after plating the cells are trypsinized with 1 ml of 0.25% trypsin-EDTA for approximately 3 minutes with rocking to remove tumor cells. To stop the action of the trypsin, 5 ml of CAF media (20% FBS/αMEM + P/S) is added and then immediately removed. The plate is washed twice with either CAF media or HBSS (no AA). Finally, 10 ml of CAF media is added to the plate and returned to the incubator to recover for 24 hours before using the cells.

### Plasmid transfection

Transient transfections were performed with either 1µg pEZX Scr vector (control), 0.5µg or 1µg pEZX miR-510 vector using the XtremeGene HP reagent as per the manufacturer’s instructions (Roche, Nutleym NJ). 48 or 72 hours after transfection, cells were harvested for protein or RNA extraction and/or assay.

### Quantitative real time PCR

Total RNA from cell lines was extracted using the RNeasyPlus Mini Kit (Qiagen; Valencia, CA). 1μg total RNA was reverse transcribed in a 20μl reaction using iScript (Bio-Rad; Hercules, CA). Real time PCR for gene expression was performed as previously described (11). Primer sequences and probe numbers are provided in **Table 1**.

**Table 1:** Primers used in the study.

Gene	UPL Number	5’ Primer Sequence	3 Primer Sequence	Amplicon Length
Human				
CAV1	42	acagcccagggaaacctc	ggatgggaacggtgtagagat	103 nt
COL1A1	50	caggcaaacctggtgaaca	ctcgccagggaaacctct	89 nt
FSP1	24	gctcaacaagtcagaactaaaggag	gcagcttcatctgtccttttc	78 nt
GAPDH	60	agccacatcgctcagacac	gcccaatacgaccaaatcc	66 nt
Mouse				
CAV1	97	aacgacgacgtggtcaaga	cacagtgaaggtggtgaagc	105 nt
COL1A1	21	cagggtcctcctggttctc	gaccgttgagtccgtctttg	124 nt
FSP1	56	ggagctgcctagcttcctg	tcctggaagtcaacttcattgtc	102 nt
GAPDH	33	aagagggatgctgcccttac	ccattttgtctacgggacga	89 nt
EPCAM	52	agaatactgtcatttgctccaaact	gttctggatcgccccttc	110 nt

### MicroRNA analysis

For microRNA analysis, 100 ng total RNA was reverse transcribed using miR-510 and U6 specific primers using the Applied Biosystems reverse transcription kit as per the manufacturer’s instructions. qPCR was performed as previously described (11).

### Western blot analysis

Cell lysate preparation and western blot analysis using enhanced chemiluminescence were performed as described previously (11). Experimental antibodies include Cav-1 (Cell Signaling Technology, 1:2500 dilution). GAPDH (Santa Cruz Technology, 1:1000 dilution) was used as a loading control. Quantitation of western blots was performed using Image J.

### CAV1 3’UTR identification and cloning

miRNA target prediction websites were mined to determine if CAV1 was a potential direct target of miR-510-5p. CAV1 3’UTR luciferase reporter vector was purchased from Genecopoeia (Rockville, MD).

### Site directed mutagenesis

The seed site within CAV1 3’UTR for miR-510-5p was mutated using a site directed mutagenesis kit (Stratagene, San Diego, CA) as per the manufacturer’s instructions. Subsequent clones were sequenced to identify those with the correct mutation.

### Dual luciferase assay

The CAV1 3’UTR luciferase reporter clone along with an empty vector (EV) control were transiently co-transfected into HEK-293 cells with and without miR-510-5p expression. Luciferase activity was measured after 48h using the dual luciferase reporter assay system (Promega, Madison, WI). Firefly luciferase activity was normalized to *Renilla* luciferase activity for ach transfected well.

### Co-culture experiments

WPMY1 fibroblasts (2.0x10^5^ cells/2mL) were seeded in the lower chamber of a 6-well dish in complete media and MDA 231 epithelial cells (2.0x10^5^ cells/1mL) either expressing miR-510-5p or a scrambled control were seeded in the upper chamber of a Transwell insert with a pore size of 0.4 µM. After 48h, the fibroblasts in the lower chamber were collected for RNA and protein analysis.

### Conditioned media experiment

WPMY1 fibroblasts were treated with 2mL/well of conditioned media from MDA 231 tumor epithelial cells.

### Transwell migration assay

WPMY1 fibroblasts (2.0x10^5^ cells/2mL) are seeded in the bottom of a 6-well dish in complete media and MDA 231 epithelial cells (2.0x10^5^ cells/1mL), either expressing miR-510-5p or a scrambled control, are seeded in the upper chamber of a Transwell insert with a pore size of 0.4 µM. After 48h, the fibroblasts in the lower chamber are collected and counted. These fibroblasts (5.0x10^4^ cells/500µL) are then seeded in serum free media in the upper chamber of a new Transwell migration insert with a pore size of 8 µM. Medium containing 10% serum is added to the lower chamber to act as a chemoattractant and incubated for 4h. Migrated cells were counted and quantified as described previously (11). Migration assays were performed in duplicate per replicate with 3 biological replicates per experiment.

### Immunohistochemistry

Immunohistochemistry staining was performed as described previously (11). Cav-1 primary antibody was used at a 1:250 dilution. 3,3′-diaminobenzidine substrate (Sigma, St Louis, MO) was added for 2 min. Slides were counterstained with hematoxylin. Five images per tumor were acquired on a Nikon 90i light microscope at 20X magnification. For Cav-1 quantitation, images were scored based on intensity of staining (0 for no staining, 1 for low staining, 2 for moderate staining, 3 for high staining, and 4 for very high staining).

### Statistical analysis

Two-sided paired Student’s *t*-tests with Welch’s correction were performed using GraphPad Prism. *p* values are given for each individual experiment, but in general, *p*< 0.05 was considered statistically significant. Error bars represent standard deviations of three independent experiments unless indicated otherwise.

## Results

### Cav1 and miR-510-5p show race specific expression in breast cancer patients

**Figure 1:**
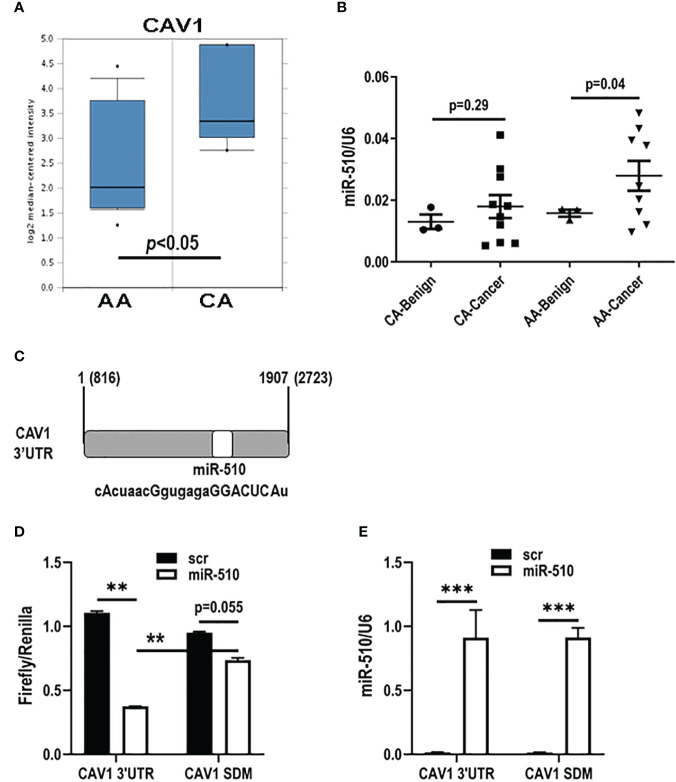
miR-510-5p directly targets CAV1 and is racially disparate in breast cancer. **(A)** Caveolin-1 (CAV1) mRNA stromal levels in African American **(**AA) and Caucasian (CA) breast cancer patients. Adapted from Chang Data set, Oncomine. **(B)** Quantitation of miR-510-5p levels from serum of AA and CA benign and cancer breast patients. **(C)** Schematic representation of miR-510-5p binding site and complementary seed sequence (upper case letters) within the 3’UTR of human CAV1. Luciferase activity **(D)** and qPCR analysis **(E)** of HEK293 cells transiently co-transfected with CAV1 3’UTR or mutated (CAV1 SDM) and miR-510-5p or scrambled control (scr). **p<0.01; ***p<0.005.

Although stromal Cav1 loss, as discussed, is associated with worse outcomes in women with breast cancer, there have been no published studies to date evaluating the role of stromal Cav1 levels in black women with breast cancer. Therefore, we performed data mining in the few publicly available data sets that are available and have stromal data and found that Cav1 mRNA levels were lower in black (AA) women with breast cancer when compared to white (CA) women in Oncomine (**Figure 1A**; Chang dataset, Oncomine) (12, 13). We have previously shown that miR-510-5p is elevated in human breast cancer tissue (14) and promotes aggressive tumor growth *in vivo* (11). Therefore, we wanted to assess whether miR-510-5p levels also differed by race. We assessed miR-510-5p by qPCR in serum samples from black (AA) and white (CA) women and found that in black women specifically, levels of miR-510-5p were elevated in women with breast cancer when compared to women with benign breast tumors (**Figure 1B**).

### Cav1 is a direct target of miR-510-5p

We performed a bioinformatical search and found that CAV1 was a predicted target for miR-510-5p [**Figure 1C**; (15, 16)]. In order to determine if CAV1 is a direct target of miR-510-5p, HEK293 cells were transiently transfected with a luciferase reporter vector containing the wild type CAV1 3’UTR or mutant CAV1 3’UTR (CAV1 SDM) with deletion of the bioinformatically predicted miR-510-5p seed site and either miR-510-5p or a scrambled control (scr). Dual luciferase assays were performed and we observed a 66% reduction in luciferase activity in the cells transfected with both the CAV1 3’UTR and miR-510-5p when compared to the scr control (**Figure 1D**). In the cells transfected with the mutant CAV1 construct, a smaller reduction (~20%) was observed in cells co-transfected with miR-510-5p compared to the scr control (**Figure 1D**). qPCR for miR-510-5p levels in the transfected cells was performed and as expected we observed significantly higher levels of miR-510-5p in the transfected HEK293 cells (**Figure 1E**). These data support CAV1 as a direct target of miR-510-5p and that this binding occurs through the predicted seed site.

### miR-510-5p expression modulates Cav-1 expression in fibroblasts

**Figure 2:**
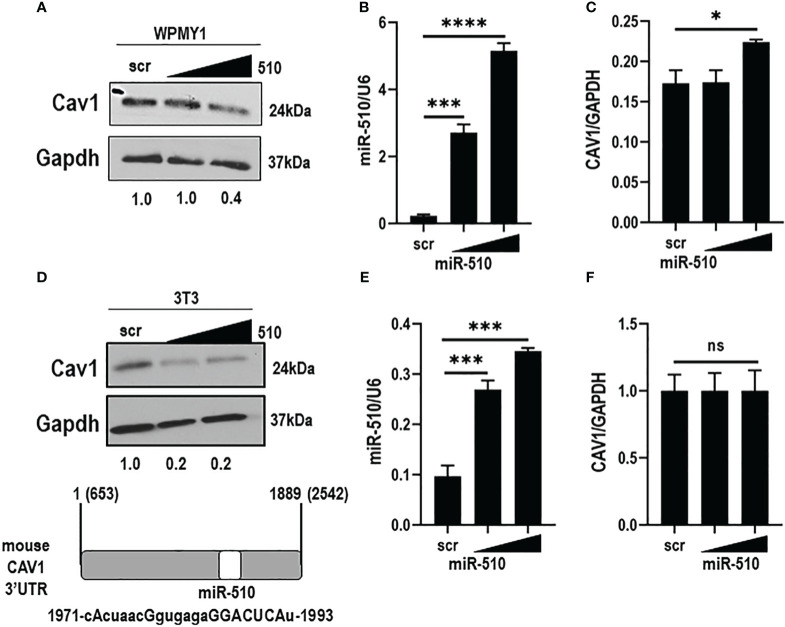
miR-510-5p inhibits Cav1 protein expression in fibroblasts. Western blot **(A)** and qPCR **(B)** analysis of Caveolin-1 and **(C)** miR-510-5p levels in human WPMY1 cells transfected with increasing concentrations of miR-510-5p or scrambled (scr) control. Western blot **(D)** and qPCR **(E)** analysis of Caveolin-1 and **(F)** miR-510-5p levels in mouse 3T3 cells transfected with increasing concentrations of miR-510-5p or scrambled (scr) control. (D: lower panel) Schematic representation of miR-510-5p binding site and complementary seed sequence (upper case letters) within the 3’UTR of mouse CAV1. *p<0.05; ***p<0.005; ****p<0.001; ns, not significant.

To assess the effects of miR-510-5p on endogenous Cav1 protein expression, western blot analysis was performed on human WPMY1 and mouse NIH3T3 stromal cell lines. Importantly, we found that the miR-510-5p seed sequence was also present within the 3’UTR of mouse CAV1, even though the miR-510-5p itself is not conserved in this species (**Figure 2D**). We observed a reduction in Cav1 protein expression levels when miR-510-5p was expressed in both WPMY1 and NIH3T3 cells (**Figures 2A, D**). qPCR for miR-510-5p levels in the transfected cells was performed as a control and as expected we observed significantly higher levels of miR-510-5p in the transfected cells (**Figures 2B, E**). We did not observe a significant decrease in Cav1 mRNA levels by qPCR in either WPMY1 or NIH3T3 cells supporting the potential mechanism of miR-510-5p mediated inhibition of CAV1 through translational repression (**Figures 2C, F**).

### Epithelial derived miR-510-5p inhibits Caveolin 1 in neighboring fibroblasts

**Figure 3:**
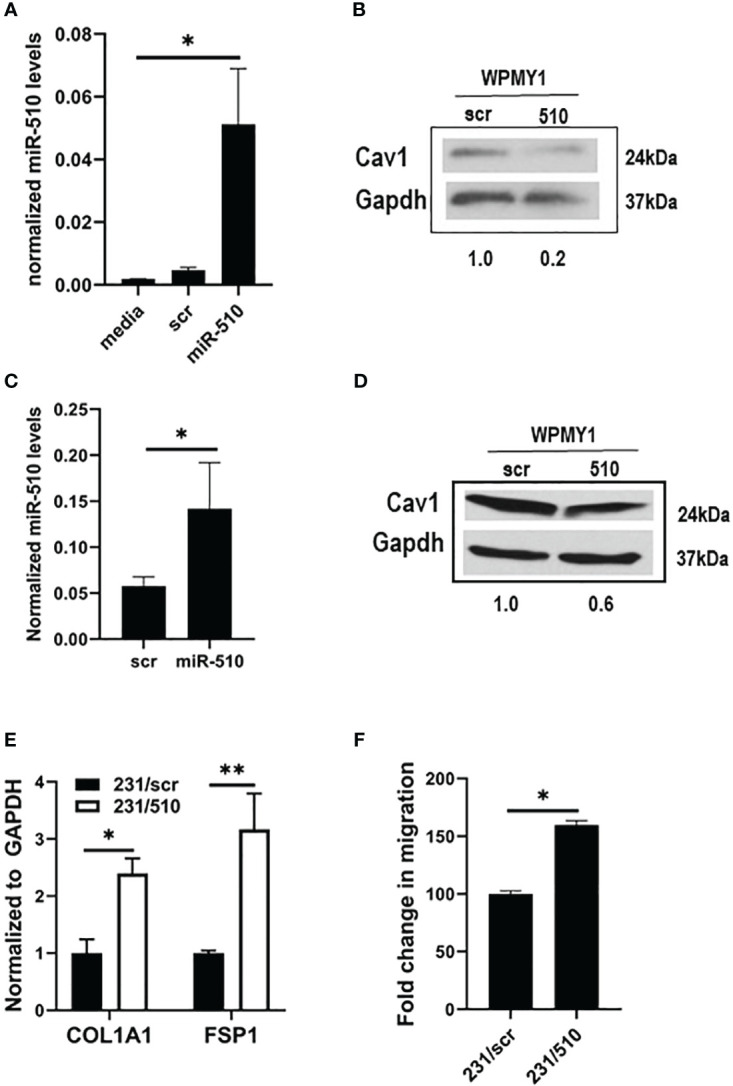
miR-510-5p activates fibroblasts and promotes cell migration. qPCR **(A)** and western blot **(B)** analysis of WPMY1 cells co-cultured with MDA 231 cells stably transfected with either miR-510-5p or scrambled (scr) control. qPCR **(C)** and western blot **(D)** analysis of WPMY1 cells treated with conditioned media from MDA 231 cells stably transfected with either miR-510-5p or scrambled (scr) control. **(E)** qPCR analysis and **(F)** Transwell migration assay of WPMY1 cells co-cultured with MDA 231 cells transfected with either miR-510-5p (231/510) or scrambled control (231/scr). *p<0.05; **p<0.01.

To examine the impact of epithelial derived miR-510-5p on stroma levels of Cav1, we performed co-culture experiments with MDA 231 epithelial cells, either stably expressing miR-510-5p or scr control, and WPMY1 fibroblasts. WPMY1 fibroblasts were collected and we performed qPCR for miR-510-5p expression. We observed a significant increase in the levels of miR-510-5p in the WPMY1 fibroblasts co-cultured with miR-510-5p expressing MDA 231 epithelial cells when compared to both the scr and media alone controls (**Figure 3A**). No significant difference was observed in WPMY1 fibroblasts co-cultured with either scr control MDA 231 epithelial cells or media alone. To determine the impact of miR-510-5p on Cav1 protein expression in WPMY1 fibroblasts we performed western blot analysis and observed a reduction in Cav1 protein in the fibroblasts that were co-cultured with miR-510-5p epithelial cells when compared to those co-cultured with scrambled control (**Figure 3B**).

To determine whether a signaling crosstalk exists between the epithelial cells and fibroblasts for this pathway to be activated, we treated WPMY1 fibroblasts for 24h with conditioned media from miR-510-5p expressing MDA 231 epithelial cells. We observed increased levels of miR-510-5p in the WPMY1 fibroblasts treated with conditioned media from miR-510-5p expressing MDA 231 epithelial cells (**Figure 3C**). We also observed a decrease in Cav1 protein levels in the WPMY1 fibroblasts treated with conditioned media from the miR-510-5p expressing MDA 231cells when compared to the scr control (**Figure 3D**).

### miR-510-5p expressing fibroblasts have an activated phenotype

To determine whether fibroblasts co-cultured with miR-510-5p overexpressing breast tumor epithelial cells are ‘activated’, we performed qPCR for collagen (COL1A1) and fibroblast specific protein (FSP1/S100A4), genes that are typically used as markers of cancer associated markers or CAFs (17). We observed a significant increase in the levels of both collagen and FSP1 in the WPMY1 fibroblasts that were co-cultured with miR-510-5p expressing MDA 231 epithelial cells when compared to the scr control (**Figure 3E**).

To determine if the ‘activated’ WPMY1 stromal fibroblasts are more aggressive compared to the non-activated scr controls, we performed a migration assay on WPMY1 fibroblasts that had been co-cultured with miR-510-5p expressing MDA 231 breast tumor epithelial cells or scr controls for 24 hours. We observed a significant increase in the migratory capacity of the WPMY1 fibroblasts from the 510-expressing epithelial co-cultures when compared to the scrambled controls (**Figure 3F**).

### miR-510-5p expressing CAFs promote aggressive tumor growth

**Figure 4:**
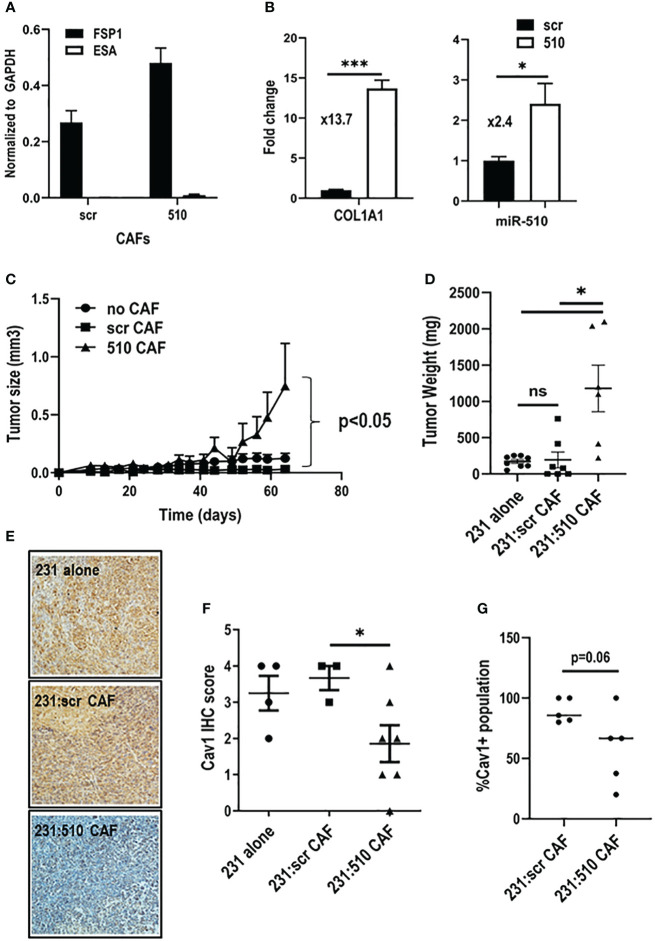
miR-510-5p expressing fibroblasts promote tumor growth. qPCR analysis of **(A)** epithelial and fibroblast markers and **(B)** COL1A1 and miR-510-5p levels in CAFs isolated from orthotopic tumors. **(C)** Tumor growth, **(D)** Tumor weight and **(F)** Cav1 quantitation of tumors resulting from orthotopic injections of MDA 231 cells either alone (2 x 10^5^) or together (1.5 x 10^5^ 231 + 5 x 10^4^ CAFs) with scr (231:scr CAF) or 510 (231:510 CAF) CAFs into female nude mice. **(E)** Representative 20X magnification images of Cav1 IHC staining. **(G)** Quantification of Cav1 protein levels in fibroblasts from orthotopic tumors. *p<0.05; ***p<0.005; ns, not significant.

To assess the impact of miR-510-5p expressing fibroblasts on epithelial tumor growth, we isolated CAFs from tumors derived from MDA 231 cells either overexpressing miR-510-5p or scr control. We performed qPCR to determine expression levels of fibroblast (FSP1) and epithelial (ESA) cell markers to ensure enriched fibroblast populations (**Figure 4A**). miR-510-5p is not conserved across all species, therefore we performed qPCR to determine if miR-510-5p was expressed in the isolated (mouse-derived) CAFs. We observed significantly increased levels of miR-510-5p in CAFs isolated from tumors with miR-510-5p overexpression (**Figure 4B**, right panel), providing further evidence for a direct transfer of miR-510-5p from tumor epithelial cells due to the lack of genomic mouse miR-510-5p. qPCR also demonstrated significantly increased expression of COL1A1 mRNA levels suggesting that these isolated fibroblasts have a more activated/aggressive phenotype (**Figure 4B**, left panel).

In order to functionally assess the impact of miR-510-5p in fibroblast mediated tumor growth, we co-injected miR-510-5p and scr derived CAFs with MDA 231 cells and assessed tumor growth. MDA 231 cells and CAFs derived from miR-510-5p and scr control tumors were injected alone to serve as additional controls. We found that the co-injection of miR-510-5p CAFs led to a significant increase in tumor growth when compared to both scr and no CAF controls (**Figures 4C, D**). There was no significant difference between the tumors derived from the MDA 231 cells alone and co-injections with scr-derived CAFs. We also performed IHC staining of tumors extracted from the mice described above and observed a significant reduction of Cav1 expression in the miR-510-5p CAF tumors when compared to both scr and no CAF controls (**Figures 4E, F**). Finally, we quantified the CAF population in the resultant tumors by immunofluorescent staining for Cav1 and found that overall there was a reduction in the amount of CAFs specifically that stained positive for Cav1 expression in the miR-510 CAF co-injected tumors (**Figure 4G**).

## Discussion

The clinical significance of Cav1 stromal loss and its associated prognostic value in breast cancer has been known for some time and yet the mechanism of its loss remains unknown. Although there are multiple studies that have demonstrated the role of miRNAs in breast cancer and both their diagnostic and prognostic value (18), there are few linking the expression of a miRNA-mediated mechanism of Cav1 loss (19, 20) and none that address the loss of Cav1 in the stromal compartment.

There remains few studies examining the role of miR-510-5p in cancer, which includes lung, pancreatic, colon and renal (21–24), and even fewer highlighting the role of miR-510-5p in breast cancer. These include our previous studies demonstrating a role for miR-510-5p in promoting aggressive tumor growth (11) and a bioinformatical study to identify noncoding RNA networks that found miR-510-5p was the most potent miRNA regulator in breast cancer using the TCGA data sets (25).

We have identified a mechanism whereby miR-510-5p upregulation in epithelial cells results in the increased expression of miR-510-5p in neighboring fibroblasts. One hypothesis is that a release of cytokines or growth factors from epithelial tumor cells leads to an upregulation of miR-510-5p in neighboring stromal cells. However, as the mouse genome does not encode for miR-510-5p, we concluded that the miR-510-5p found in CAFs from our *in vivo* experiments must have originated from the human epithelial cells that were injected. Therefore, we propose that miR-510-5p is transported from epithelial cells to the fibroblasts either in an extracellular vesicle, like an exosome, or as a vesicle-independent Ago-2 associated miRNA and will be the subject of future studies.

Fibroblasts are one of the most abundant cell types within the tumor microenvironment, and once activated are known to promote aggressive tumor growth. This study shows that miR-510-5p expression in fibroblasts leads to an activated phenotype, specifically with the upregulation of collagen. In the context of Cav1 loss and breast cancer this is relevant as studies have shown that Cav1-/- stromal cells, a genetic model of activated myofibroblasts (8) secrete more collagen. In addition, in human breast cancer patients, fibrosis is a risk factor for poor clinical outcome (4). The field of CAFs is burgeoning with different subsets of CAFs being identified in more recent years with emerging technologies like single cell RNA Seq becoming more readily available. A consensus currently exists whereby four main subsets of CAFs have been identified to exist in breast cancer (26). Subsets 1 and 4 are the predominant types that exist with subset 1 playing a role in immune suppression. Future studies will involve a more comprehensive analysis of the type of CAF subset that we observe with miR-510-5p expression.

No studies have examined the levels of Cav1 in terms of breast cancer disparities. A study in prostate cancer showed that epithelial expression of Cav1 was higher in black men when compared to white men (27). This study is the only paper that we are aware of to examine Cav1 expression as a cancer disparity and is highly relevant due to the studies showing that an inverse correlation exists between epithelial and stromal Cav1 expression in cancer tissues (24), therefore one may extrapolate from this that stromal expression of Cav1 would be lower in black men with prostate cancer. Similarly, our work supports the idea that Cav1 levels are lower in the stroma of black women with breast cancer. Significantly, our data showed that miR-510-5p levels are significantly elevated in black women with breast cancer compared to benign controls. Although the number of samples was small, this difference was not observed in the white breast cancer patients. In summary, these data support a novel microRNA-mediated mechanism of Cav1 reduction in breast cancer patients and warrants future studies exploring it role in promoting breast cancer disparities.

## Data availability statement

The raw data supporting the conclusions of this article will be made available by the authors, without undue reservation.

## Ethics statement

The studies involving humans were approved by IRB at the Medical University of South Carolina. The studies were conducted in accordance with the local legislation and institutional requirements. The human samples used in this study were acquired from de-identified samples from the Hollings Cancer Center Tissue Biorepository. Written informed consent for participation was not required from the participants or the participants’ legal guardians/next of kin in accordance with the national legislation and institutional requirements. The animal study was approved by IACUC at the Medical University of South Carolina.

## Author contributions

AE-K, SH, DPT, and VJF contributed to conception and design of the study. BK, QG, BB, BAK, GJ and AS performed the *in vitro* experiments. LMN and BK performed the *in vivo* experiments. BK, QG, and BAK performed the statistical analysis. VJF wrote the first draft of the manuscript. DPT and SH wrote sections of the manuscript. All authors contributed to the article and approved the submitted version.
